# Determining Predictors of Early Response to Exenatide in Patients with Type 2 Diabetes Mellitus

**DOI:** 10.1155/2015/162718

**Published:** 2015-01-20

**Authors:** Muhammad Khan, Jing Ouyang, Karen Perkins, Sunil Nair, Franklin Joseph

**Affiliations:** Department of Diabetes & Endocrinology, Countess of Chester Hospital NHS Foundation Trust, Chester CH2 1UL, UK

## Abstract

Exenatide is a GLP-1 analogue used in the management of T2DM yet within a subset of patients fails due to adverse side effects or from failure to attain the end goal. This retrospective observational study aimed to determine whether we could predict response to exenatide in patients with T2DM. 112 patients on exenatide were included with patient age, gender, duration of T2DM, medications alongside exenatide and weight, BMI, and HbA1c at baseline and 3 and 6 months of exenatide use being recorded. 63 responded with 11 mmol/mol reduction from baseline HbA1c after six months and 49 did not respond to exenatide. HbA1c solely differed significantly between cohorts at baseline, 3 months, and 6 months (*P* < 0.05). Regression analyses identified a negative linear relationship with higher baseline HbA1c correlating to greater reductions in HbA1c by 6 months (*P* < 0.0001). HbA1c was the sole predictor of exenatide response with higher baseline HbA1c increasing the odds of response by 5% (*P* = 0.004). Patients with HbA1c reductions ≥15–20% by 3 months were more likely to be responders by 6 months (*P* = 0.033). Our study identified that baseline HbA1c acted as the sole predictor of exenatide response and that response may be determined after 3 months of exenatide administration.

## 1. Introduction

Exploiting the incretin effect [1, 2], glucagon-like peptide-1 (GLP-1) analogues are primarily utilised for their glucose lowering abilities through stimulation of insulin in a glucose-dependent manner [3, 4]. Nevertheless, GLP-1 analogues provide additional benefits which include the control of glucose excursions by attenuating the rate of gastric emptying, inhibition of glucagon secretion, and promotion of weight loss by augmenting the sensation of satiety [5, 6]. Most promisingly, GLP-1 analogues promote the proliferation, survival, and neogenesis of pancreatic *β*-cells, a facet not shared by existing antihyperglycaemic agents [7–9]. With such beneficial properties, GLP-1 analogues are commonplace in the glycaemic control of patients with type 2 diabetes mellitus (T2DM) and presently four drugs within this class are licensed for use in T2DM: exenatide (Byetta) [10], liraglutide (Victoza) [11], lixisenatide (Lyxumia) [12], and prolonged-release exenatide (Bydureon) [13].

As established by the National Institute of Health and Care Excellence (NICE), GLP-1 analogues are utilised as a third-line therapy when both first-line metformin and second-line sulfonylureas fail to provide adequate glycaemic control despite appropriate diet and exercise interventions [10]. To date, several clinical trials have demonstrated their efficacious ability to induce weight loss and improve glycaemic control in type 2 diabetic patients [14–24]. Nonetheless, within such studies, GLP-1 analogues also fail in a cohort of individuals due to adverse effects associated with these agents (primary failure) or by not achieving the defined end-goal (secondary failure).

Although the definition of primary failure is standardised between healthcare institutes, different healthcare systems adhere to differing “end-goal” definitions. Within the UK, NICE define an individual who is said to have “responded” to therapy if their baseline weight and glycated haemoglobin (HbA1c) have reduced by 3% and an 11 mmol/mol (1%), respectively, after six months of GLP-1 analogue administration [10]. If such criteria have been satisfied GLP-1 analogue therapy is continued whereas if not the next appropriate therapy, typically insulin, is considered. Despite the required weight loss ensuring that GLP-1 analogues are used cost-effectively, an 11 mmol/mol (1%) drop in HbA1c is known to reduce the development of microvascular complications, such as diabetic retinopathy, nephropathy, and neuropathy, by 25% [25]. However, limited information is currently available which delineates factors that predict whether individuals will respond or not to GLP-1 analogues. At present, the few studies in this area of research have identified that baseline HbA1c appears to be the strongest predictor of response to GLP-1 analogues [26–31]. Nevertheless, such studies are hindered by both small sample sizes and a lack of comparisons at multiple time points. Furthermore, identification of individuals unlikely to respond to GLP-1 analogues early on would nullify the theoretical risk of exposing patients to a 6-month period of side-effects, ranging from nausea and vomiting to acute pancreatitis [14–17], and the significant expense associated with GLP-1 analogues without any clinical benefit [10]. Therefore, the possibility of differentiating between responders and nonresponders to GLP-1 analogues based upon patient characteristics and/or changes in anthropometric and metabolic parameters remains a topical subject amongst clinicians.

Subsequently, the primary aim of our study was to determine whether we could identify, in a group of patients with T2DM initiated on the GLP-1 analogue exenatide, any predictors of response by identifying any differences between parameters in those that achieved the NICE required reduction in HbA1c (responders) and those that did not (nonresponders). Additionally, our secondary aim was to translate any identified predictors of glycaemic response into a quantifiable figure, whereby this figure could be used to distinguish between individuals who would then achieve the targeted HbA1c reduction from those that would not.

## 2. Methods

This retrospective observational study was registered and conducted at the Countess of Chester NHS Foundation Trust (CoCH), Cheshire, United Kingdom. Participants were identified following review of patients with diabetes mellitus who were initiated on a GLP-1 analogue from 2008 to 2014 at the Endocrinology Department at the CoCH. Medical records for participants fulfilling the inclusion criteria were then accessed using the programme MEDITECH (Medical Information Technology, Massachusetts). Data collected included the patient's age, gender, and the number of years diagnosed with T2DM, concurrent diabetic medications, and the patient's weight, body mass index (BMI), and HbA1c at baseline (date initiated on exenatide), 3 months, and 6 months after exenatide initiation. All variables explored in this study were included in the audit protocol, thereby conforming to the ethical standards and framework set by the CoCH.

### 2.1. Inclusion and Exclusion Criteria

Patients were included if they met the following criteria: diagnosis of T2DM made using the clinical criteria established by the NICE [10], ≥18 years of age, initiated on exenatide, and required data available at baseline, 3 months, and 6 months after exenatide therapy. Exenatide was chosen as the sole GLP-1 analogue under analysis due to it being the most prescribed GLP-1 analogue at the CoCH and our experience with this agent in clinical practice. Additionally, prolonged-release exenatide was not considered due to the increased time period required for this drug to be efficacious; thus our defined time points would not necessarily reflect any glycaemic benefit. Patients were excluded if diagnosed with other subsets of diabetes mellitus (DM), such as type 1 diabetes mellitus (T1DM) and gestational diabetes, exposure to previous GLP-1 analogue treatment, prescribed GLP-1 analogues that did not include exenatide, prescribed weight reducing medications alongside exenatide, and if complete data was not available at the aforementioned time points.

### 2.2. Study Cohorts

Participants who satisfied the inclusion criteria were divided into two groups: responders and nonresponders. Responders refer to individuals whose baseline HbA1c fell by the NICE instigated ≥11 mmol/mol (1%) reduction after 6 months of exenatide use, whereas nonresponders categorise patients who failed to achieve this decrease. The additional 3% weight loss requirement also stated by NICE [10] was not included in the response definition, as the 25% reduction in microvascular risk associated with a ≥11 mmol/mol reduction in HbA1c is, in our opinion, of greater clinical importance. Furthermore, we also categorised another group of participants as those with primary failure to exenatide treatment. This group included individuals that experienced side effects within the first two weeks of initiating exenatide resulting in the participant discontinuing further treatment.

### 2.3. Statistical Analyses

Results obtained from the study are presented as mean ± standard deviation (SD) or as percentages (%) for continuous and categorical variables, respectively. The significance of any differences between mean values obtained from both responders and nonresponders was determined using the independent Student's *t*-test for continuous data and Chi-Square test or Fisher's Exact for categorical data; Fisher's Exact test was used if sample size within categories was less than 5. To determine if any changes observed in weight, BMI, or HbA1c over the three time points for the entire cohort, responder group, and nonresponder group were of statistical significance, repeated measures analysis of variance (RM-ANOVA) was used. If statistical significance was observed, Tukey's test was used to correct for multiple comparisons. In order to develop a model that could predict and define the relationship between statistically varying variables and attainment of the defined HbA1c reduction, both linear regression and binary logistic regression analysis were used. Results were deemed statistically significant if the *P* value was <0.05 and all statistical analyses were conducted using the statistical software package GraphPad Prism 6.

## 3. Results

391 patients were identified for possible inclusion within this study. 253 individuals were excluded on the basis of incomplete data (*n* = 206), prescribed a GLP-1 analogue other than exenatide (*n* = 46; liraglutide = 31; lixisenatide = 7; prolonged-release exenatide = 8), or diagnosed with T1DM (*n* = 1). 26 patients were further excluded following primary failure due to nausea and vomiting. This left 112 participants available for statistical analyses with 63 responding and 49 not responding to exenatide, as defined by a reduction ≥11 mmol/mol (1%) in HbA1c from baseline to six months. None of the 112 participants reported any adverse effects and none reported any symptomatic hypoglycaemia.

Initial comparisons of patient demographics between responders and nonresponders identified no significant differences in gender (percentage male), age of participants within the cohorts, and duration of T2DM (Table 1). Additionally, differences in medications prescribe alongside exenatide were similar between the two groups with the exception of the number prescribed exenatide with insulin. Nonresponders were shown to have a significantly greater proportion of participants on exenatide with insulin compared to responders (20.4 versus 3.17%, *P* = 0.0047).

Weight for the entire cohort reduced from 113 ± 17.9 kg at baseline to 109 ± 18.1 kg at 3 months and 106 ± 18.9 kg at 6 months (*P* < 0.0001). Similarly, the BMI for the entire cohort reduced from 39.2 ± 5.85 kg/m^2^ at baseline to 37.7 ± 5.91 kg/m^2^ at 3 months and 36.8 ± 6.31 kg/m^2^ at 6 months (*P* < 0.0001). HbA1c across the study period reduced from a baseline value of 80.9 ± 14.6 mmol/mol to 70.4 ± 13.4 mmol/mol by 3 months and 67.8 ± 14.9 mmol/mol at 6 months (*P* < 0.0001). Analysis of these changes between time points showed it to be statistically significant (*P* < 0.0001).

For both responders and nonresponders, there was a significant reduction in both weight and BMI from baseline to six months (*P* < 0.001) (Table 2). Although responders had a higher baseline weight compared to nonresponders (114 ± 18.6 versus 111 ± 16.3 kg) (39.4 ± 5.63 versus 38.4 ± 5.7 kg/m^2^), both groups achieved the same mean weight by 3 months (106 ± 20.6 versus 106 ± 16.8 kg) and responders had a lower BMI by 6 months compared to nonresponders (36.5 ± 6.57 versus 37.1 ± 6 kg/m^2^) (Table 2).

Comparisons in the HbA1c change within responders identified a reduction from 85.1 ± 15.4 mmol/mol at baseline to 67.8 ± 13.4 mmol/mol by 3 months and 60.7 ± 12.2 mmol/mol by 6 months (*P* < 0.001). However, nonresponders demonstrated an initial reduction in HbA1c by 3 months (73.7 ± 12.9 mmol/mol) from baseline (75.5 ± 11.4 mmol/mol), yet by 6 months the HbA1c had risen to a level greater than baseline (77 ± 13.2 mmol/mol). Although the overall change in the nonresponders was shown to be significant (*P* = 0.028), following correction for multiple comparisons, it was observed that the only statistically significant difference was the elevation in HbA1c from 3 months to 6 months (*P* = 0.032) (Table 2).

**Figure 1:**
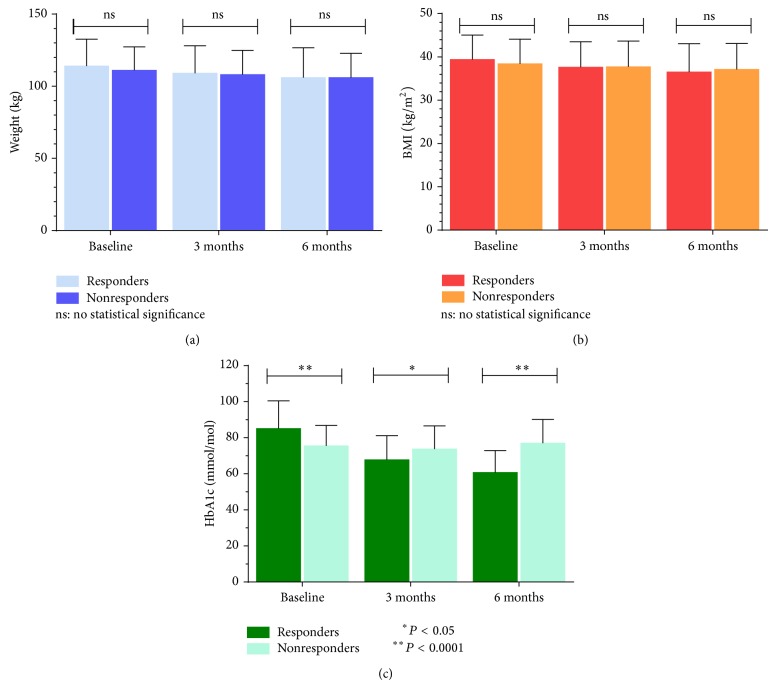
Mean (±1 SD) changes in weight (a), BMI (b), and HbA1c (c) between responders (*n* = 63) and nonresponders (*n* = 49) at baseline, 3 months, and 6 months. NS denotes no statistical significance between groups. Asterisks denote statistical significance where ∗∗ is a *P* value <0.0001 and ∗ is a *P* value <0.05. Abbreviations: BMI, body mass index; HbA1c, glycated haemoglobin.

Analysis of weight between responders and nonresponders identified no significant differences between the two cohorts at baseline (114 ± 18.6 versus 111 ± 16.3 kg, *P* = 0.54), 3 months (109 ± 19.1 versus 108 ± 16.9 kg, *P* = 0.86), and 6 months (106 ± 20.6 versus 106 ± 16.8 kg, *P* = 0.83) (Figure 1(a)). Similarly, any differences in the BMI between responders and nonresponders at baseline (39.4 ± 5.63 versus 38.4 ± 5.7 kg/m^2^), 3 months (37.6 ± 5.91 versus 37.7 ± 5.97 kg/m^2^), and 6 months (36.5 ± 6.57 versus 37.1 ± 6 kg/m^2^) were also not significant with *P* values of 0.75, 0.92, and 0.61, respectively (Figure 1(b)). However, the HbA1c recorded for responders and nonresponders was shown to significantly differ at baseline (85.1 ± 15.4 versus 75.5 ± 11.4 mmol/mol, *P* < 0.001), 3 months (67.8 ± 13.4 versus 73.7 ± 12.9 mmol/mol, *P* = 0.021), and 6 months (60.7 ± 12.2 versus 77 ± 13.2 mmol/mol, *P* < 0.001) (Figure 1(c)).

**Figure 2:**
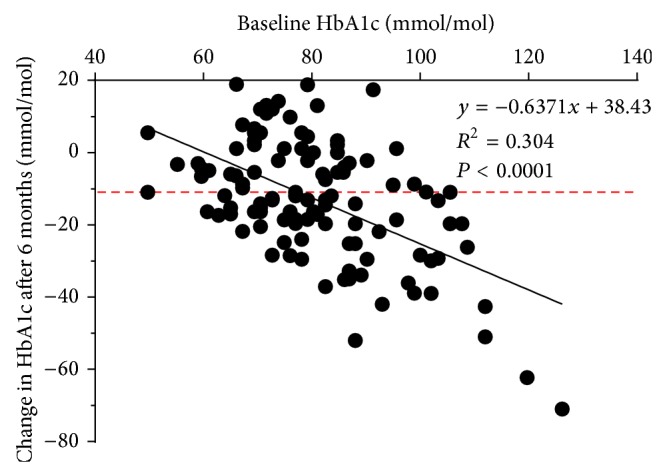
Relationship between the baseline HbA1c and changes in HbA1c after six months of exenatide therapy. Results are shown with the equation for the line of best fit, the *R* ^2^ value quantifying the goodness of fit, and the *P* value showing statistical significance. Dashed line indicates the 11 mmol/mol point which is the reduction required for individuals to be classed as responders to exenatide. Abbreviations: HbA1c, glycated haemoglobin.

Linear regression analysis identified a significant relationship between the baseline HbA1c and changes in HbA1c from baseline to 6 months (*P* < 0.0001), with the *x*-intercept at 60.3 ± 14 mmol/mol (Figure 2). Therefore, individuals with a baseline HbA1c <60.3 mmol/mol were not likely to respond, yet those with a baseline HbA1c ≥60.3 mmol/mol were more likely to respond to exenatide therapy over the 6-month period. Additionally, those with a higher baseline HbA1c experienced a greater relative and absolute reduction in their HbA1c over the study period. No significant relationship was observed for any other variable (*P* > 0.05).

Of the several variables included within the binary logistic regression, only baseline HbA1c predicted response as defined by a reduction in 11 mmol/mol after 6 months of exenatide therapy (Table 3). Our model demonstrates that individuals with a higher baseline HbA1c have a 5% increased odds of being classed as responders to exenatide by 6 months (*P* = 0.004). Although our model identified exenatide with insulin therapy as increasing the odds of responding compared to exenatide alone, the wide confidence interval range makes this variable unlikely to predict response to exenatide therapy.

In order to determine the change in HbA1c required for response to exenatide therapy, we quantified the percentage difference in HbA1c from baseline to 3 months against the proportion of participants responding and not responding to exenatide by 6 months (Table 4). Comparisons within categories revealed that a reduction of 15–20% in HbA1c by 3 months compared to baseline was the first statistically significant difference that resulted in a greater proportion of responders compared to nonresponders (*P* = 0.033). Therefore, this would indicate that if the patient's baseline HbA1c decreases within a range of ≥15–20% (or more) by 3 months, they are more likely to achieve the 11 mmol/mol reduction in HbA1c by 6 months. Furthermore, a reduction in the HbA1c from baseline between 0 and 5% resulted in the first significant difference that resulted in a greater proportion of nonresponders compared to responders. Subsequently, those who have a reduction in HbA1c between 0 and 5% (or less) by 3 months are more likely to not reach the 11 mmol/mol reduction in HbA1c by 6 months.

## 4. Discussion

The clinical benefits of exenatide as a monotherapy or in combination with either existing oral antihyperglycaemic agents (OHAs) and/or insulin have been extensively documented elsewhere [14–17]. Our study reflects similar improvements as exemplified by the significant reduction observed overall in the mean HbA1c, weight, and BMI across the entire cohort of participants. Therefore, our study reinforces the utilisation of exenatide as a GLP-1 analogue in the management of T2DM and additionally emphasises the efficacious properties of this GLP-1 analogue with regard to improvements in both weight and glycaemic control.

However, within the aforementioned studies there is a significant proportion of the initial cohort that either experience side-effects preventing them from continuing exenatide (primary failure) or fail on the basis of not achieving the defined end-point (secondary failure). Our study reflects this phenomenon as demonstrated by 18.8% of the initial cohort discontinuing therapy following primary failure due to intolerable nausea and vomiting. Additionally, of the participants that did complete 6 months of exenatide therapy, 43.8% failed to achieve the required 11 mmol/mol reduction in HbA1c stipulated by NICE [10]. Such observations reflect those reported by the limited literature presently available with studies reporting primary failure rates within the range of 11.4–33.6%, in addition to secondary failure rates ranging from 39 to 61% [26, 27, 29–31]. Although the rates of both primary and secondary failure rates observed within our study lie within such ranges, it reiterates the clinical significance and need of identifying measures which can delineate whether an individual will respond or not respond to exenatide therapy. By doing so, those distinguished as unlikely to benefit from exenatide therapy can be initiated on the next most suitable therapeutic adjunct appropriate for managing their type 2 diabetes, thus reducing the cost and exposure to side effects associated with exenatide with no clinical benefit.

Within our study, levels of HbA1c were the only parameter to significantly differ between responders and nonresponders with our regression analysis identifying that higher levels of HbA1c at baseline were associated with greater reductions in HbA1c over the study period. Such findings have been reported in the majority of the literature within this field [26, 27, 30, 31]; however, Song et al. also noted significant correlations between changes in HbA1c and the patient's age, levels of serum fasting glucose, and parameters that measure pancreatic-*β* cell function such as homeostatic model assessment-*β* (HOMA-*β*), levels of insulin at baseline, and both basal and stimulated C-peptide levels [30]. As we were unable to measure variables of insulin secretion, we cannot ascertain whether such a relationship in the changes in HbA1c and these parameters would have existed in our study. However, with regard to age, no other study has identified such a relationship and despite the study by Shin et al. also including cohorts of South Korean type 2 diabetics only, they did not report such an association either [31]. Therefore, this association may simply be exclusive to the study sample used by Song et al. [30].

Additionally, our predictive model noted that baseline HbA1c was the sole indicator of response to exenatide therapy with higher baseline values of HbA1c corresponding to 5% greater odds of exenatide response by 6 months. Although several studies have reported additional parameters as predictors of response, the most reproducible variable has been baseline HbA1c. However, within the existing literature the predictive role of baseline HbA1c determining response to exenatide is shown to differ between studies. Both Anichini et al. and Preumont et al. report that a higher baseline HbA1c reduces the odds of response [27, 29], yet studies by Anderson et al. and Shin et al. observed a greater likelihood of response with higher levels of baseline HbA1c [26, 31]. Although results from our study correspond to the latter studies, we believe the differing predictive role of baseline HbA1c observed by Preumont et al. and Anichini et al. is a result of the definition used to determine response to exenatide. Both Preumont et al. and Anichini et al. defined response to exenatide as participants achieving an absolute HbA1c value ≤58 mmol/mol (7.5%) [27] and HbA1c ≤53 mmol/mol (7%), respectively [29]. However, in both our study and those conducted by Anderson et al. and Shin et al., response to exenatide was defined by a relative loss in HbA1c [26, 31]. Subsequently, the variations in the predictive value of baseline HbA1c may be in part attributed to whether an absolute or a relative value was used as the response definition. One explanation for this is that despite individuals with a higher baseline HbA1c reporting a greater relative reduction in their HbA1c, it is more difficult for these individuals to reach the absolute HbA1c response cut-off due to the large reduction required to reach that point. In contrast, those with a baseline HbA1c that marginally differs from the HbA1c response value are, despite having only a relatively small reduction in their HbA1c, more likely to reach this end-point. We believe this policy of defining response based upon attainment of an absolute value should not be used to define response to exenatide, but rather a relative reduction. Studies such as the UK Prospective Diabetes Study (UKPDS) have shown that an 11 mmol/mol (1%) reduction in HbA1c reduces the risk of microvascular complications by 25% [25]; thus it would be clinically unwise to omit individuals with a high baseline HbA1c from receiving exenatide despite knowing these are more likely to show a significant relative reduction overall.

In our study we also identified patients with a baseline HbA1c <60.3 mmol/mol (7.7%) as unlikely to respond to exenatide, whereas those with an HbA1c at baseline ≥60.3 mmol/mol (7.7%) were likely to respond. Such findings have been reported by Anderson et al. which noted that individuals with a baseline HbA1c <56.3 mmol/mol (7.3%) were less likely to respond to exenatide compared to those with a baseline HbA1c ≥56.3 mmol/mol (7.3%) [26]. The significance of these observations correlates to the requirements for use of exenatide as defined by NICE [10]. NICE state that exenatide should be utilised when both first-line metformin and second-line sulphonylureas have failed, in addition to the patients' HbA1c being ≥58.5 mmol/mol (7.5%) [10]. Subsequently, our results coincide with such criteria for exenatide use as patients with a baseline HbA1c <60.3 mmol/mol had no response to exenatide; thus clinicians should avoid utilising exenatide in such scenarios as it only exposes patients to the possible side effects and the financial implications associated with exenatide with no improvement in glycaemic control.

Although identifying individuals with a baseline HbA1c value <60.3 mmol/mol (7.7%) served as one means of delineating nonresponse to exenatide therapy, it only accounted for 12.2% of the cohort that eventually failed to respond following 6 months of exenatide administration. Unlike the previous studies within this area, we are the only study to utilise three time-points, that is, baseline, 3 months, and 6 months. Therefore, analyses in the percentage change in HbA1c at 3 months compared to baseline identified another measure of predicting response to exenatide therapy. In our study, which we believe is the first to report such an observation, a reduction in the baseline HbA1c by 15–20% (or greater) at the 3-month period resulted in a greater proportion of the cohort following on to be classed as responders at 6 months. Furthermore, individuals who have had an HbA1c reduction between 0 and 5% (or less) at 3 months resulted in a significantly greater proportion being deemed as nonresponders by 6 months. At present, NICE guidelines advocate determining response to exenatide following 6 months of use [10]. It is only at this point that clinicians are deemed appropriate to make a judgement regarding whether an individual is suitable to continue exenatide therapy and if not, an alternative therapy will then be commenced which will also be monitored to observe for any glycaemic benefit. This attitude within healthcare to periodically observe and in some situations fail to alter or adequately titrate upwards treatment strategies in the face of an uncontrolled disease is referred to as therapeutic inertia [32]. In our study we identified that a significant proportion of the initial cohort could be identified as responders by the 3-month period simply by quantifying the percentage difference in HbA1c from baseline. Therefore, in the clinical setting clinicians would only have to wait for 3 months to determine whether an individual was likely to respond, thus allowing for the rapid intensification or alteration of treatment if shown to be not working whilst also ameliorating a further three-month period of exposure to the theoretical side effects and cost attributed to exenatide.

During the course of this study we encountered limitations which may have influenced our results. Firstly, we did not envisage such a large proportion of the identified sample to be excluded on the basis of incomplete data. This in turn led to a reduced sample size available for statistical analyses and thus may have resulted in fewer variables being identified as predictors of response. Secondly, all our participants were derived from one institute and so this puts into question whether our predictive model is applicable to other institutes. Furthermore, the patients we included within our study were identified from a cohort under the care of the endocrine department at the CoCH, thereby exposing our study to selection bias. As such patients in our clinics reflect individuals with difficult cases of T2DM, and not the typical cases seen in primary care, inclusion of such cases m ay have skewed our results to reflect a predictive model only applicable for such treatment resistant cases. Another limitation of our study is that we did not measure adherence to exenatide during the study period. As exenatide is associated with both side effects and the possibility of stigma arising due to use of injections, patients may have not adhered to the agreed upon treatment plan. This limitation may be responsible for the significant rise in HbA1c identified between 3 months and 6 months in the nonresponder group. Additionally, in our study we cannot ascertain whether the weight loss observed is a direct consequence of exenatide suppressing appetite or as a result of concurrent dietary and lifestyle interventions undertaken by the patient. Finally, despite identifying other medications prescribe alongside exenatide, we did record the initial dosage and whether medication regimens changed over the course of the study. Therefore, this may have meant that any observed changes may have been, in part, attributed to the additional medications given alongside exenatide and not exenatide alone.

Although our study provides another dimension, applicable to the real world setting that clinicians should consider when determining if an individual will respond to exenatide, we believe additional research examining the other GLP-1 analogues is warranted; there is currently only one study on predictors of response to liraglutide [28]. Furthermore, it would be of clinical relevance to determine if the predictors of response to exenatide differ depending upon inclusion of the 3% weight to our existing definition of response to exenatide.

## 5. Conclusion

Patients with T2DM represent a diverse heterogeneous population with varying demographic, anthropometric, and metabolic characteristics. Despite exenatide being shown to be efficacious, there remains a subset of individuals who fail to respond. Our results reinforces what has been documented previously with regard to the negative linear relationship between baseline HbA1c and changes in HbA1c but additionally sheds further insight into the conflicting nature of baseline HbA1c as a predictor of response. Furthermore, our study provides a novel insight into the possibility of using the percentage change in HbA1c observed at 3 months of treatment to predict response by 6 months. We hope that our findings reinvigorate this field of research as with supplementary research we can ultimately develop a predictive model which both outlines and quantifies a series of predictor variables and changes allowing clinicians to identify, with a significant degree of confidence, individuals likely to respond to exenatide therapy.

## Figures and Tables

**Table 1:** Comparison between responders (*n* = 63) and nonresponders (*n* = 49) in patient demographics and medications prescribed alongside exenatide. Results are presented as the mean ± SD for continuous variables and as the number (plus percentage) for categorical variables.

Variable	Group		*P* value
	Responders	Nonresponders	
Gender male (% male)	36 (57)	28 (57)	1.00
Age	59 ± 11	61 ± 10	0.16
Duration of T2DM in years	14 ± 6.06	14.9 ± 5.83	0.40
Prescribed exenatide only (%)	5 (7.94)	2 (4.08)	0.46
Prescribed exenatide with OHAs only (%)	45 (71.4)	27 (55.1)	0.11
Prescribed exenatide with insulin only (%)	**2 (3.17)**	**10 (20.4)**	**0.0047**
Prescribed exenatide with OHAs and insulin (%)	11 (17.5)	10 (20.4)	0.88

OHA: oral antihyperglycaemic agent; T2DM: type 2 diabetes mellitus.

**Table 2:** Mean (±SD) weight, BMI, and HbA1c at baseline, 3 months, and 6 months within responders (*n* = 63) and nonresponders (*n* = 49). The overall *P* value reflects the significance before adjustment and adjusted *P* value denotes the significance between time points following Tukey's test for multiple comparisons.

Parameter	Responders			Overall *P* value	Adjusted *P* value		
	Baseline	3 months	6 months		Baseline to 3 months	Baseline to 6 months	3 months to 6 months
Weight (kg)	114 ± 18.6	109 ± 19.1	106 ± 20.6	<0.001	<0.001	<0.001	<0.001
BMI (kg/m^2^)	39.4 ± 5.63	37.6 ± 5.91	36.5 ± 6.57	<0.001	<0.001	<0.001	<0.001
HbA1c (mmol/mol)	85.1 ± 15.4	67.8 ± 13.4	60.7 ± 12.2	<0.001	<0.001	<0.001	<0.001

Parameter	Nonresponders			Overall *P* value	Adjusted *P* value		
	Baseline	3 months	6 months		Baseline to 3 months	Baseline to 6 months	3 months to 6 months

Weight (kg)	111 ± 16.3	108 ± 16.9	106 ± 16.8	<0.001	<0.001	<0.001	<0.001
BMI (kg/m^2^)	38.4 ± 5.7	37.7 ± 5.97	37.1 ± 6	<0.001	<0.001	<0.001	<0.001
HbA1c (mmol/mol)	75.5 ± 11.4	73.7 ± 12.9	77 ± 13.2	0.028	0.29	0.42	0.032

BMI: body mass index; HbA1c: glycated haemoglobin.

**Table 3:** Results from the binary logistic regression identifying variables which can predict response to exenatide therapy.

Variable	Odds ratio	95% confidence intervals	*P* value
Age	0.977	0.942–1.01	0.20
Gender^*^	1.07	0.502–2.26	0.87
Duration of diabetes (years)	0.972	0.913–1.04	0.38
Baseline weight (kg)	1.00	0.986–1.03	0.50
Baseline BMI (kg/m^2^)	1.02	0.951–1.08	0.66
Baseline HbA1c (mmol/mol)	**1.05**	**1.02–1.08**	**0.004**
OHA only^**^	1.48	0.267–8.17	0.66
Insulin only^**^	**8.33**	**1.03–67.1**	**0.046**
OHAs and insulin^**^	2.50	0.397–15.7	0.33

∗ denotes a variable that was compared against females; ∗∗ denotes a variable that was compared against exenatide as a monotherapy. BMI: body mass index; HbA1c: glycated haemoglobin; OHA: oral antihyperglycaemic agent.

**Table 4:** Contingency table quantifying the percentage change in HbA1c after 3 months of exenatide therapy from baseline against the proportion of participants who went on to respond and not to respond to exenatide by 6 months.

		Number of responders and nonresponders		*P* value
		Responders	Nonresponders	
Difference in HbA1c after 3 months of exenatide therapy compared to baseline HbA1c (%)	*x* > 5	0	10	0.0001
	0 < *x* ≤ 5	2	6	0.13
	0	2	3	0.65
	**−5 ≤ ** **x** ** < 0**	**4**	**10**	**0.041**
	−10 ≤ *x* < −5	7	9	0.29
	−15 ≤ *x* < −10	7	6	0.85
	**−20 ≤ ** **x** ** < −15**	**13**	**3**	**0.033**
	−25 ≤ *x* < −20	11	2	0.037
	−25 < *x*	17	0	0.0001

*x* denotes the percentage difference. A reduction in HbA1c from baseline after 3 months of exenatide therapy is indicated by a − value. HbA1c: glycated haemoglobin.

## Abbreviations

DM: Diabetes mellitus
HbA1c: Glycated haemoglobin
BMI: Body mass index
GLP-1: Glucagon-like peptide 1
NICE: National Institute for Health and Care Excellence
CoCH: Countess of Chester Hospital
UK: United Kingdom.
